# Pain relief but respiratory depression: Insights into the dark side of opioid overuse

**DOI:** 10.1113/JP292044

**Published:** 2026-08-04

**Authors:** Daniel B. Zoccal

**Affiliations:** ^1^ Department of Physiology and Pathology, School of Dentistry of Araraquara São Paulo State University (UNESP) Araraquara Brazil

**Keywords:** brainstem, breathing, opioid

Opioids are commonly used for acute pain management owing to their potent peripheral and central effects in suppressing nociceptive transmission and processing. Their analgesic effect results primarily from activation of μ‐opioid receptors (μORs), with synthetic agonists being more potent than natural opiates (for instance, fentanyl is 70–80 times more potent than morphine). Despite their pain‐relieving benefits, opioids can cause serious side effects, including constipation, suppressed immune function, tolerance and use disorder. The issue of addiction is particularly important because opioids are also used indiscriminately to produce euphoria, pleasure and emotional comfort (Lambert, 2023). Evidence shows that overuse of opioids for both medical and recreational purposes, combined with their addictive properties, has had devastating global consequences, with opioid‐related deaths rising exponentially. Opioid‐induced respiratory depression (OIRD) is the main cause of death associated with excessive opioid use. It involves slow, irregular and shallow breathing, which can progress to prolonged apnoea or respiratory arrest in severe cases (Ramirez et al., 2021). The disruptive effects of opioids on breathing control systems are also mediated by the activation of μORs, and treatment with opioid receptor antagonists, such as naloxone, can reverse these respiratory symptoms. However, this treatment also eliminates the pain‐relieving effects, resulting in severe pain and withdrawal symptoms. Therefore, there is a clear need for new experimental and preclinical studies investigating the cellular and molecular mechanisms underlying OIRD.

Breathing emerges from interconnected rhythmic networks in the brainstem that comprise excitatory and inhibitory neurons, with a specialized excitatory core population in the preBötzinger complex (preBötC) that is indispensable for respiratory rhythm and pattern formation. μORs are expressed throughout the brainstem respiratory network, causing hyperpolarization of presynaptic and postsynaptic terminals mainly by suppressing voltage‐gated calcium channels or by opening potassium channels (Ramirez et al., 2021). Therefore, understanding the effects of opioids on the respiratory network and deciphering the underlying mechanisms of OIRD is not a simple task. In a recent elegant publication in *The Journal of Physiology*, Kalajian et al. (2026) provided detailed evidence on the cellular and ionic mechanisms behind opioid‐induced suppression of the preBötC neuronal activity. Although other respiratory regions contribute to OIRD (Varga et al., 2020), the preBötC has been studied extensively as a critical site of opioid action because of its essential role in rhythm generation. This region contains inhibitory and excitatory interneurons, some of which express μORs and possess distinct ion channels that influence their firing activity, including various potassium channels (Picardo et al., 2013), thereby making the understanding of opioid effects here more complex.

Kalajian et al. (2026) combined neuroanatomical and refined electrophysiological studies in genetically modified animals to interrogate excitatory (glutamatergic) and inhibitory (GABAergic and glycinergic) preBötC neurons selectively and to characterize the ionic mechanism modulated by opioids. In adult animals, the authors identified that *Oprm1* mRNA (transcripts associated with μORs) is present in both excitatory and inhibitory neurons within the preBötC region. By performing whole‐cell and single‐channel patch‐clamp recordings in brainstem slices of neonatal animals, the authors found that DAMGO, a μOR agonist, activated Kir3 (G‐protein‐coupled inwardly rectifying potassium, GIRK) channels via membrane‐delimited signalling, thereby increasing potassium conductance in preBötC neurons. This functional effect was restricted to excitatory neurons, because inhibitory neurons were insensitive to DAMGO, and it did not involve other relevant potassium channels expressed in the preBötC neurons. This study, therefore, provides direct and detailed evidence of the cellular substrate and the postsynaptic ion channel effector modulated by the downstream opioid signalling in the preBötC.

It is clear that the findings reported by Kalajian et al. (2026) enhance our understanding of opioid effects on respiratory control mechanisms, particularly in the preBötC. Also important, the findings of this study prompt additional questions regarding this topic and how opioids might affect other bodily systems. For instance, the observation that inhibitory neurons in neonates were insensitive to DAMGO, despite the presence of μOR mRNA in inhibitory neurons in adult animals, raises questions about age‐related differences in μOR expression or post‐transcriptional mechanisms between excitatory and inhibitory populations of the preBötC, which might affect the mechanisms underlying OIRD in neonates and adults. Furthermore, the experiments and analyses by Kalajian et al. (2026) might have encompassed additional populations of inhibitory, non‐respiratory neurons within the ventral medulla that overlap the preBötC region, including neurons in the caudal ventrolateral medulla involved in the regulation of sympathetic activity. If so, these findings raise the intriguing possibility that opioids engage additional ventromedullary neural circuits that control homeostatic functions beyond respiratory rhythm generation, particularly in adult animals. Although addressing these questions was beyond the scope of the study by Kalajian et al. (2026), it represents an important avenue for future investigation.

The technically rigorous study by Kalajian et al. (2026) provides important insights into the cellular substrates and ionic mechanisms within the core respiratory rhythm generator that are modulated by opioids, enhancing our understanding of their depressant effects on respiration. Furthermore, by identifying a membrane‐delimited signalling pathway involving G‐protein βγ subunits that is modulated by opioids in the preBötC, it is tempting to speculate that interventions targeting these mechanisms could yield new therapies for OIRD while avoiding some of the inherent limitations of conventional strategies. However, translation of these findings into clinical treatments will require preclinical and clinical validation.

## Additional information

### Competing interests

None declared.

### Author contributions

Sole author.

### Funding

The author thanks the São Paulo Research Foundation (FAPESP, 2025/05255‐4) and the National Council for Scientific and Technological Development (CNPq, 303481/2021‐8; 408542/2025‐0) for financial support.

### Generative AI statement

No generative artificial intelligence tools were used in the preparation of this manuscript.

## Supporting information


Peer Review History
