# Supplementary material for: Pain relief but respiratory depression: Insights into the dark side of opioid overuse
Source: J Physiol. 2026 Aug 4;604(17):7017–8. doi: 10.1113/JP292044 (PMC13532945; doi:10.1113/JP292044)
Supplement: Supplementary file 1 [file TJP-604-7017-s001.pdf]

The Journal of Physiology

Pain relief but respiratory depression: insights into the dark side of opioid overuse

Daniel B Zoccal
DOI: 10.1113/JP292044

Corresponding author(s): Daniel Zoccal (daniel.zoccal@unesp.br)

The following individual(s) involved in review of this submission have agreed to reveal their identity: Christopher A Del Negro (Referee #1)

Review Timeline:

Submission Date:	16-Jul-2026
Editorial Decision:	18-Jul-2026
Revision Received:	20-Jul-2026
Accepted:	22-Jul-2026

Senior Editor: Kim Barrett

Reviewing Editor: Vaughan Macefield

Transaction Report:

(Note: With the exception of the correction of typographical or spelling errors that could be a source of ambiguity, letters and reports are not edited. Depending on transfer agreements, referee reports obtained elsewhere may or may not be included in this compilation. Referee reports are anonymous unless the Referee chooses to sign their reports.)

Dear Daniel,

Re: JP-P-2026-292044 "Pain relief but respiratory depression: insights into the dark side of opioid overuse" by Daniel B Zoccal

Thank you for submitting your manuscript to The Journal of Physiology. It has been assessed by a Reviewing Editor and by 1 expert referee and we are pleased to tell you that it is acceptable for publication following satisfactory revision.

Please advise your co-authors of this decision as soon as possible.

The review comments are copied at the end of this email.

Please address all the points raised and incorporate all requested revisions or explain in your Response to Referees why a change has not been made. We hope you will find the comments helpful and that you will be able to return your revised manuscript within 2 weeks. If you require longer than this, please contact journal staff: jp@physoc.org.

Your revised manuscript should be submitted online using the link in your Author Tasks Link Not Available. This link is accessible via your account as Corresponding Author; it is not available to your co-authors. If this presents a problem, please contact journal staff (jp@physoc.org). Image files from the previous version are retained on the system. Please ensure you replace or remove any files that are being revised.

If you do not wish to submit a revised version of your manuscript, you must inform our journal staff (jp@physoc.org) or reply to this email to request withdrawal. Please note that a manuscript must be formally withdrawn from the peer review process at one journal before it may be submitted to another journal.

TRANSPARENT PEER REVIEW POLICY: To improve the transparency of its peer review process The Journal of Physiology publishes online, as supporting information, the peer review history of all articles accepted for publication. Readers will have access to decision letters, including Editors' comments and referee reports, for each version of the manuscript, as well as any author responses to peer review comments. Referees can decide whether or not they wish to be named on the peer review history document.

LANGUAGE EDITING AND SUPPORT FOR PUBLICATION: If you would like help with English language editing, or other article preparation support, Wiley Editing Services offers expert help, including English Language Editing, as well as translation, manuscript formatting, and figure formatting at www.wileyauthors.com/eeo/preparation. You can also find resources for Preparing Your Article for general guidance about writing and preparing your manuscript at www.wileyauthors.com/eeo/prepresources.

REVISION CHECKLIST:

Upload a full Response to Referees file. To create your 'Response to Referees' copy all the reports, including any comments from the Senior and Reviewing Editors, into a Microsoft Word, or similar, file and respond to each point, using font or background colour to distinguish comments and responses and upload as the required file type.

Please upload two versions of your manuscript text: one with all relevant changes highlighted and one clean version with no changes tracked. The manuscript file should include all tables and figure legends, but each figure/graph should be uploaded as separate, high-resolution files. The journal is now integrated with Wiley's Image Checking service. For further details, see: https://www.wiley.com/en-us/network/publishing/research-publishing/trending-stories/upholding-image-integrity-wileys-image-screening-service

You may also upload:
- 'Potential Cover Art' for consideration as the issue's cover image
- Appropriate Supporting Information (Video, audio or data set: see https://jp.msubmit.net/cgi-bin/main.plex?form_type=display_requirements#supp).

We look forward to receiving your revised submission.

If you have any queries, please reply to this email and we will be pleased to advise.

Best wishes,

Kim Barrett
Senior Editor
The Journal of Physiology

EDITOR COMMENTS

Reviewing Editor:

Thank you for submitting your Perspectives article to The Journal of Physiology, which has been assessed by the authors of the manuscript to which you refer. As you will see, the authors have a few small recommendations I would like you to attend to, and look forward to receiving your revised manuscript shortly.

Senior Editor:

Thanks for going above and beyond to author this nice Perspectives article, Daniel!

REFEREE COMMENTS

Referee #1:

The Perspective understands the highlighted paper quite well. I have a few respectful suggestions for the author of the piece.

In paragraph 2, the author refers to the "complex neural network in the brainstem, consisting or interconnected clusters of excitatory and inhibitory neurons, each of which plays a distinct and significant role in shaping respiratory pattern and regulating rhythmic activity." This is factually true, of course, and accurate, but excessively wordy in my view. I think it may overwhelm the newcomer to research in the field because of "complexity" and "distinct roles". I think it would suffice to write "Breathing is generated by rhythmic interconnected networks in the brainstem, which features excitatory and inhibitory neurons but with a specialized excitatory core population in the preBötzinger complex (preBötC), which is indispensable for rhythm and pattern." That would put the focus of the piece on the preBötC a bit earlier than it is now. At present, the preBötC is introduced after referencing Kalajian et al., whereas I think (respectfully) it may be better to introduce the preBötC as the core oscillator beforehand.

In paragraph 3, Kalajian et al. used RNAscope for neuroanatomy, but not Imaging in the conventional sense, i.e., Calcium imaging. Same line, I recommend omitting "cellular" from "cellular electrophysiological studies" because -- as the author writes a little later -- we also used single-channel recordings, which are not "cellular" per se.

Paragraph 4 discusses some of the important caveats of the study, for example, why do inhibitory neurons express mu-opioid receptors (at least in adult mice), but not respond to opioid drugs (at least in neonates), which is excellent.

However, there is something in the Kalajian study that (I think) could be mentioned to intrigue the reader. Kalajian et al. point out that the membrane-delimited signaling pathway, via G-beta-gamma subunits, might provide a pharmacological target to combat opioid-induced respiratory depression. That type of strategy avoids the limitations of naloxone, including re-narcotization and low potency compared to synthetic opioids. I respectfully encourage the author to mention that point of the paper too. Perhaps something like "In demonstrating that the opioid modulation occurs via a membrane-delimited signaling pathway, the authors identify potentially druggable targets that might be able to combat OIRD while avoiding some of the inherent limitations of conventional strategies like Narcan, which affect all signaling pathways (not just potassium channels) and can be outmatched by synethetic opioids with stronger affinity for the receptor and longer half-life than Narcan."

END OF COMMENTS

JP-P-2026-292044 – R1

Response to the Editors' comment

Senior Editor: Thanks for going above and beyond to author this nice Perspectives article, Daniel!

Reviewing Editor: Thank you for submitting your Perspectives article to The Journal of Physiology, which has been assessed by the authors of the manuscript to which you refer. As you will see, the authors have a few small recommendations I would like you to attend to, and look forward to receiving your revised manuscript shortly.

Author's response: I thank the Editors for their positive feedback and suggestions on the perspective article. I appreciate the opportunity to submit a revised version and to address the Referee's comments, as detailed below.

Response to the Referee's comments

Referee: The Perspective understands the highlighted paper quite well. I have a few respectful suggestions for the author of the piece.

Author's response: I thank the Referee for his thoughtful feedback and valuable suggestions.

Referee: In paragraph 2, the author refers to the "complex neural network in the brainstem, consisting or interconnected clusters of excitatory and inhibitory neurons, each of which plays a distinct and significant role in shaping respiratory pattern and regulating rhythmic activity." This is factually true, of course, and accurate, but excessively wordy in my view. I think it may overwhelm the newcomer to research in the field because of "complexity" and "distinct roles". I think it would suffice to write "Breathing is generated by rhythmic interconnected networks in the brainstem, which features excitatory and inhibitory neurons but with a specialized excitatory core population in the preBötzinger complex (preBötC), which is indispensable for rhythm and pattern." That would put the focus of the piece on the preBötC a bit earlier than it is now. At present, the preBötC is introduced after referencing Kalajian et al., whereas I think (respectfully) it may be better to introduce the preBötC as the core oscillator beforehand.

Author's response: I thank the Referee for providing his opinion on the text. I agree that the text can be revised to make it more accessible to new readers. The sentence highlighted by the Referee was modified.

Referee: In paragraph 3, Kalajian et al. used RNAscope for neuroanatomy, but not Imaging in the conventional sense, i.e., Calcium imaging. Same line, I recommend omitting "cellular" from "cellular electrophysiological studies" because -- as the author writes a little later -- we also used single-channel recordings, which are not "cellular" per se.

Author's response: I thank the Referee for pointing out these details. The text was revised accordingly.

Referee: Paragraph 4 discusses some of the important caveats of the study, for example, why do inhibitory neurons express mu-opioid receptors (at least in adult mice), but not respond to opioid drugs (at least in neonates), which is excellent. However, there is something in the Kalajian study that (I think) could be mentioned to intrigue the reader. Kalajian et al. point out that the membrane-delimited signaling pathway, via G-beta-gamma subunits, might provide a pharmacological target to combat opioid-induced respiratory depression. That type of strategy avoids the limitations of naloxone, including re-narcotization and low potency compared to synthetic opioids. I respectfully encourage the author to mention that point of the paper too. Perhaps something like "In demonstrating that the opioid modulation occurs via a membrane-delimited signaling pathway, the authors identify potentially druggable targets that might be able to combat OIRD while avoiding some of the inherent limitations of conventional strategies like Narcan, which affect all signaling pathways (not just potassium channels) and can be outmatched by synethetic opioids with stronger affinity for the receptor and longer half-life than Narcan."

Author's response: I thank the Referee for this important suggestion. The last paragraph of the article was revised to incorporate the Referee's point of view while preserving the original tone.

Dear Dr Zoccal,

Re: JP-P-2026-292044R1 "Pain relief but respiratory depression: insights into the dark side of opioid overuse" by Daniel B Zoccal

We are pleased to tell you that your paper has been accepted for publication in The Journal of Physiology.

The last Word (or similar) version of the manuscript provided will be used by the Production Editor to prepare your proof. When this is ready you will receive an email containing a link to Wiley's Online Proofing System. The proof should be thoroughly checked and corrected as promptly as possible.

Authors should note that it is too late at this point to offer corrections prior to proofreading. Major corrections at proof stage, such as changes to figures, will be referred to the Editors for approval before they can be incorporated. Only minor changes, such as to style and consistency, should be made at proof stage. Changes that need to be made after proof stage will usually require a formal correction notice.
All queries at proof stage should be sent to: TJP@wiley.com.

Following copyediting and typesetting the accepted version will be published online.

Please note that Perspective articles are not typically covered by institutional open access agreements with our publisher, Wiley. Wiley do not offer article processing charge (APC) discounts for smaller article types in hybrid subscription journals, meaning that if you wish for your Perspective to be published Open Access, you will have to pay the full APC. As such, we recommend authors publish Perspectives 'behind the paywall', where they will become freely accessible after a 12-month embargo (i.e. please select the NON open access option via 'Wiley Authors' during proofing).

Should you wish to pay for Open Access, you will be able to place an order by logging into Wiley Author services.

• You can check if your funder or institution has a Wiley Open Access Account here:
https://authors.wiley.com/author-resources/Journal-Authors/open-access/author-compliance-tool.html

Yours sincerely,

Kim Barrett
Senior Editor
The Journal of Physiology

IMPORTANT POINTS TO NOTE FOLLOWING ACCEPTANCE OF YOUR PAPER:

• IMPORTANT NOTICE ABOUT OPEN ACCESS: To assist authors whose funding agencies mandate immediate public access to published research findings, The Journal of Physiology allows authors to pay an Open Access (OA) fee to have their papers made freely available immediately on publication.

The Corresponding Author will receive an email from Wiley with details on how to register or log in to Wiley Authors where you will be able to place an order.

• You can check if your funder or institution has a Wiley Open Access Account here: https://authors.wiley.com/author-resources/Journal-Authors/open-access/author-compliance-tool.html

MEMBER DISCOUNT: Corresponding authors who are Members of the Physiological Society are eligible for a 20% discount on the cost of the Article Processing Charge (APC). To claim this discount, the Corresponding Author will need to log in to the Physiological Society Membership portal and retrieve the discount code (https://www.physoc.org/journals-and-media/journals/journal-discount-for-members/). This code should then be quoted in the billing section of Wiley Authors when selecting the open access option.

• TRANSPARENT PEER REVIEW POLICY: To improve the transparency of its peer review process, The Journal of Physiology publishes online as supporting information the peer review history of all articles accepted for publication. Readers will have access to decision letters, including Editors' comments and referee reports, for each version of the manuscript as well as any author responses to peer review comments. Referees can decide whether or not they wish to be named on the peer review history document.

• You can help your research get the attention it deserves! Check out Wiley's free Promotion Guide for best-practice recommendations for promoting your work at: www.wileyauthors.com/eeo/guide. You can learn more about Wiley Editing Services which offers professional video, design, and writing services to create shareable video abstracts, infographics,

conference posters, lay summaries, and research news stories for your research at: www.wileyauthors.com/eeo/promotion.

• If you would like to receive our 'Research Roundup', a monthly newsletter highlighting the cutting-edge research published in The Physiological Society's family of journals (The Journal of Physiology, Experimental Physiology, Physiological Reports, The Journal of Nutritional Physiology and The Journal of Precision Medicine: Health and Disease), please click this link, fill in your name and email address and select 'Research Roundup':
https://www.physoc.org/journals-and-media/membernews

EDITOR COMMENTS

Reviewing Editor:

Thank you for attending to the remaining minor concerns.
